# Effect of Adipose-Derived Mesenchymal Stem Cells (ADMSCs) Application in Achilles-Tendon Injury in an Animal Model

**DOI:** 10.3390/cimb44120396

**Published:** 2022-11-22

**Authors:** Ángel Arnaud-Franco, Jorge Lara-Arias, Iván A. Marino-Martínez, Oscar Cienfuegos-Jiménez, Álvaro Barbosa-Quintana, Víctor M. Peña-Martínez

**Affiliations:** 1 Facultad de Medicina, Servicio de Ortopedia y Traumatología, Universidad Autónoma de Nuevo León, Monterrey 66451, Nuevo León, Mexico; 2 Centro de Investigación en Ciencias de la Salud (CIDICS), Universidad Autónoma de Nuevo León, Monterrey 66451, Nuevo León, Mexico; 3 Departamento de Patología, Facultad de Medicina, Universidad Autónoma de Nuevo León, Monterrey 66451, Nuevo León, Mexico; 4 Facultad de Medicina, Servicio de Anatomía Patológica y Citopatología, Universidad Autónoma de Nuevo León, Monterrey 66451, Nuevo León, Mexico

**Keywords:** ADMSCs, Achilles tendon, tendinopathy, stem cell therapy

## Abstract

Background: Achilles-tendon rupture prevails as a common tendon pathology. Adipose-derived mesenchymal stem cells (ADMSCs) are multipotent stem cells derived from adipose tissue with attractive regeneration properties; thus, their application in tendinopathies could be beneficial. Methods: Male rabbit ADMSCs were obtained from the falciform ligament according to previously established methods. After tenotomy and suture of the Achilles tendon, 1 × 10^6^ flow-cytometry-characterized male ADMSCs were injected in four female New Zealand white rabbits in the experimental group (ADMSC group), whereas four rabbits were left untreated (lesion group). Confirmation of ADMSC presence in the injured site after 12 weeks was performed with quantitative sex-determining region Y (SRY)-gene RT-PCR. At Week 12, histochemical analysis was performed to evaluate tissue regeneration along with quantitative RT-PCR of collagen I and collagen III mRNA. Results: Presence of male ADMSCs was confirmed at Week 12. No statistically significant differences were found in the histochemical analysis; however, statistically significant differences between ADMSC and lesion group expression of collagen I and collagen III were evidenced, with 36.6% and 24.1% GAPDH-normalized mean expression, respectively, for collagen I (*p* < 0.05) and 26.3% and 11.9% GAPDH-normalized mean expression, respectively, for collagen III (*p* < 0.05). The expression ratio between the ADMSC and lesion group was 1.5 and 2.2 for collagen I and collagen III, respectively. Conclusion: Our results make an important contribution to the understanding and effect of ADMSCs in Achilles-tendon rupture.

## 1. Introduction

Anatomically and functionally, the Achilles tendon, also called the calcaneal tendon, is the thickest and largest in the human body. This tendon is involved in the movement of three joints, namely knee flexion, plantar flexion, and hindfoot inversion. The primary function of the Achilles tendon is to serve as an attachment structure between bones and muscles, and due to its anatomy and location between the calcaneus and calf muscles in the lower extremity, the tendon must be capable of withstanding high stretch tension caused by the traction of the muscles on the heel. To fulfill this function, at the cellular and molecular level, tenocytes have motor proteins such as actin and myosin [1], which allow the tendon tissue to have properties of motor transmissibility, as well as resistance to high tensile forces. It is estimated that the tendons have the capacity to stretch up to 4% of their length before being damaged [2], with the Achilles tendon capable of withstanding a tensile force of more than 3 kN [3].

Achilles-tendon injuries are prevalent conditions that range from inflammatory pathologies such as tendinitis, to tissue rupture, and can be broadly classified into acute injuries and chronic injuries, as well as insertional and noninsertional tendinopathies. The incidence of Achilles tendinosis is generally unknown. However, an incidence of up to 18% in runners, 9% in dancers, 5% in gymnasts, 2% in tennis players, and less than 1% in football players has been reported [4]. It is estimated that around 1 million athletes suffer from a tendon injury per year [5].

Moreover, there are certain extrinsic factors that can contribute to the weakening of the tendon, such as drugs (steroids and fluoroquinolones) [6] and intrinsic factors such as age, gender, and obesity [7].

Achilles-tendon injuries that do not involve rupture usually have a favorable prognosis and recovery assuming correct physiotherapeutic management and, where appropriate, treatment of the underlying disease in those related to rheumatic diseases (e.g., enthesitis due to spondyloarthropathies). However, the total or partial rupture of the tendon usually brings with it sequelae that affect the quality of life of the patient, in particular the loss of mobility and strength [8,9].

Conventional treatment for Achilles-tendon rupture includes open surgery, percutaneous surgery, and conservative treatment. Although generally efficient and with a low rate of rerupture, open and percutaneous surgery have been associated with sural nerve injuries that can compromise the functionality of the limb [10], while conservative treatment, although not associated with iatrogenic injuries, on the other hand, has been associated with reruptures of the tendon [11].

In addition to conventional methods for Achilles-tendon repair such as surgery and conservative treatment, there are strategies based on biological agents that seek to stimulate the healing capacity of injured tissue, such as platelet-rich plasma (PRP), growth factors, hydrogels, gene therapy, and cell therapy with mesenchymal stem cells (MSC), from which benefits such as increased strength and early functional restoration have been found [12,13,14,15].

Mesenchymal stem cells (MSCs) are multipotent cells that have interesting properties for regenerative therapy such as injured-tissue migration, immunomodulation, and repair. Nonetheless, MSCs are easy to differentiate into various cell types, maintaining their proliferative capacity in vitro [16]. Such cells can be isolated from bone marrow (BMMSC), umbilical cord (UCMSC), and bloodstream and adipose tissue (ADMSC) with ease [17]. The regenerative capacity of these cells has been attributed especially to the secretion of growth factors and anti-inflammatory agents [18,19]. In preclinical studies, MSCs have been shown to be useful in the management of tendinitis in horses [20]; in addition, treatment with BMMSCs has been shown to be superior in the treatment of Achilles-tendon rupture in a rat model compared to PRP, according to histological, biochemical, and immunohistochemical parameters [21]. In particular, the use of ADMSCs has been frequent due to the ease of isolating these cells from adipose tissue. Moreover, their relevance in regenerative medicine has been increasing, being applied in a wide variety of tissues [22,23,24]. Thus, based on the scientific evidence, the present work intends to evaluate the healing effect of the administration of ADMSC directly applied in a previously injured Achilles tendon of New Zealand rabbits.

## 2. Materials and Methods

### 2.1. Study Design

The present work is an experimental, cross-sectional, comparative, controlled, and blind study with respect to the evaluator in an animal model.

### 2.2. Ethics and Animal Welfare

Prior approval of the Ethics Committee, Internal Committee for the Care and Use of Laboratory Animals (CICUAL), and the Biosafety Committee of the Faculty of Medicine of the Autonomous University of Nuevo León with the registration number OR14-001. Throughout the trial, it was ensured that the rabbits could move freely within the cage. They were kept on food and clean water. In order to perform the tenotomies, all rabbits were administered analgesia and antibiotic therapy. Sacrifice was performed by sedation in accordance with the Official Mexican regulation NOM-062-ZOO-1999 (Technical Specifications for the Production, Care, and Use of Laboratory Animals).

### 2.3. Group Description

In the present study, we used twelve healthy adult rabbits of three months of age and 2.5 kg on average, four of which were males and eight were females. Animal models were divided into three groups.

ADMSC group—Cell treatment. Four female New Zealand white rabbits underwent percutaneous Achilles-tendon tenotomy with fixation at both ends; 1 × 10^6^ cells were applied in the injured area.

Lesion group—Injured control. Four female New Zealand white rabbits underwent percutaneous Achilles-tendon tenotomy with fixation at both ends and no treatment.

Healthy group—Four male New Zealand white rabbits without any intervention.

Male rabbits of this group were also adipose tissue donors.

### 2.4. Extraction of ADMSC from Donor Rabbits, Cell Culture, and Flow Cytometry Characterization

The adipose tissue samples obtained from the liver falciform ligament of male New Zealand white rabbits were processed in a class II laminar flow hood (SterilGARD^®^ III Advance. THE BAKER COMPANY, Gatehouse Road. Sanford, FL, USA). The adipose tissue was placed in a sterile 50 mL Falcon tube and washed with PBS buffer supplemented with gentamicin in order to remove as much blood and stromal tissue as possible.

The adipose tissue was placed in a flask with a magnetic stirrer inside and was resuspended in 20 mL of collagenase type I (GIBCO-BRL LIFE TECHNOLOGIES, Grand Island, NY, USA) at 0.1% for digestion of the stromal matrix. At the end of the enzymatic digestion time, 2 volumes of PBS were added in order to form two phases with the adipose tissue at the top and cells at the bottom. The lower phase was recovered and the washing process with PBS was repeated on two more occasions to recover as many cells as possible. The cells were cultured for two weeks in DMEM supplemented with 10% FBS in order to obtain enough cells to cover the dosage needs for the ADMSC group.

For flow cytometry analysis, an average of 3 × 10^6^ cells were used, which were resuspended in 50 μL of staining buffer (SB, Becton Dickinson Pharmingen). 10 μL of each antibody was added at a 1:10 dilution and incubated for 20 min (Table 1). Samples were washed with 1 mL of 1× PBS and centrifuged at 2000 rpm for 10 min at 4 °C. The stained cells from each tube were resuspended in 250 μL of 1× PBS and passed through the flow cytometer (BD LSRFortessa^TM^, Franklin Lakes, NJ, USA).

### 2.5. Percutaneous Tenotomy

New Zealand white female rabbits were intramuscularly anesthetized with Acepromazine (0.5 mg/kg), Ketamine (50 mg/kg), and Xylazine (5 mg/kg) in order to perform the percutaneous tenotomy. Vital signs were monitored during the procedure. Subsequently, a catheter was placed in the marginal vein to supply tramadol (5 mg/kg) and to maintain sedation if necessary. Both groups underwent asepsis of both legs in the area of the Achilles tendon, and percutaneous tenotomy was performed with a #15 scalpel blade making a 3 mm incision, taking the rabbit’s ankle in maximum dorsiflexion until the rupture of the Achilles tendon was felt. This procedure was performed bilaterally and both ends were sutured with Vicryl 3-0 to prevent the retraction of both ends of the tendon. Finally, the wound was sutured with 3-0 nylon.

### 2.6. ADMSC Administration

In order to obtain a suspension of ADMSCs, cell cultures were incubated with Trypsin/EDTA 0.25% for 5 min. Then, 1× PBS washings were performed, and cells were counted to obtain 1 × 10^6^ prior to 10 min centrifugation at 1500 rpm. Cells were resuspended in 250 μL of PBS. The 250 μL solution of ADMSCs was injected into the ADMSC group directly in the lesion area. The rabbits were maintained with food and water ad libitum until reaching 12 weeks after cell treatment, established as the sacrifice moment.

### 2.7. Histopathological Analysis

The tissue changes were evaluated at Week 12 post-cell administration.

Masson’s trichrome and H&E staining were performed to identify the organization of collagen fibers.

The Grande Histological Biomechanical Correlation Score (GHBCS), described by Andrew et al. [18], was employed in order to compare histological results in surgically repaired tendon, rating collagen orientation, angiogenesis, and induction for cartilage formation.

The analysis was carried out with three blinded pathologist observers, who graded the tissue under observation corresponding to the groups previously described.

### 2.8. Molecular Analysis

DNA extraction was performed from biopsies taken from both groups at the injured area using Triton X-100 2%, SDS 1%, NaCl, 100 mM, Tris HCI 10 mM pH 8, and EDTA 1 mM pH 8. To locate the cells of the male rabbits (donors), an endpoint PCR was performed using markers previously reported in the gene bank to identify the sex-determining region Y (SRY) gene (Accession Number; AY785433), with the following primer sequences; Forward: AGC GGC CAG GAA CGG GTC AAG, Reverse: CCT TCC GGC GAG GTC TGT ACT TG). GAPDH was used as an endogenous gene control (Accession Number: L23961) with the following primer sequences; Forward: TGA ACG GAT TTG GCC GCA TTG, Reverse: ATG CCG AAG TGG TCG TGG ATG). PCR conditions were the following: 98 °C for 30 s, 92 °C for 1 s, 64 °C for 35 s, 35 cycles with extension at 72 °C for 1 min (Eppendorf 5331 MasterCycler Gradient Thermal Cycler, Eppendorf, Hamburg, Germany). The reaction volume was 15 μL, which contained 20 mM ammonium sulfate, 75 mM Tris-HCl pH 8.8, 0.1% Tween 20, 1.5 mM MgCl_2_, 0.4 μM of primers, 250 μM dNTPs, and 1U Platinum Taq DNA Polymerase (Invitrogen^TM^ Thermo Fisher Scientific, Waltham, MA, USA). The amplified product was separated on 2% agarose gel containing EtBr at 180 mA in 10 mM lithium borate buffer, pH 8.0, for 15 min (Sigma-Aldrich, St. Louis, MA, USA). The products were visualized under UV light with the UVP VisiDoc-It™ 100 imaging system (Analytik Jena, Upland, CA, USA) camera. In order to quantify the expression of collagens I and III, an RT-qPCR was performed using mRNA obtained with TRIzol reagent from the isolated cells using primer sets D49399 (Forward: TTC TGC AGG GCT CCA ATG A, Reverse: TCG ACA AGA ACA GTG TAA GTG AAC CT, TaqMan probe: TTG AAC TTG TTG CCG AGG GCA ACA G) for collagen I and S83371 for collagen III (Forward: CCT GAA GCC CCA GCA GAA, Reverse: AAC AGA AAT TTA GTT GGT CAC TTG TAC TG, TaqMan probe: TTG CAC ATT TTA TAT GTG TTC CTT TTG TTC TAA TCT TGT C). GAPDH quantification was performed with the following primer sequences; Forward: TGC ACC ACC ACC AAC TGC TTA G, Reverse: GGT CTT CTG GGT GGC AGT GTG A, TaqMan Probe: TCA TCC ACG ACC ACT TCG GCA TTG T and used as a housekeeping gene. Expression levels were normalized to GAPDH using the delta-delta Ct method. We used the formula 2^−ΔCt^ to obtain normalized fold gene expression values relative to GAPDH for each of the four evaluated animals per group. Then, the mean of this relative fold gene expression value for each group was obtained and expressed as a percentage relative to the housekeeping gene. Finally, the expression ratio between the experimental (ADMSC) and control (Lesion) groups (2^−ΔΔCt^) was calculated. 

### 2.9. Statistical Analysis

The Mann–Whitney U test was used to compare two independent samples. The results were considered statistically significant if *p* < 0.05 was reached. Mean, interquartile range (IQR), and standard error of the mean (SEM) were carried out with the SPSSV24 package along with the aforementioned Mann–Whitney U test.

## 3. Results

**Figure 1:**
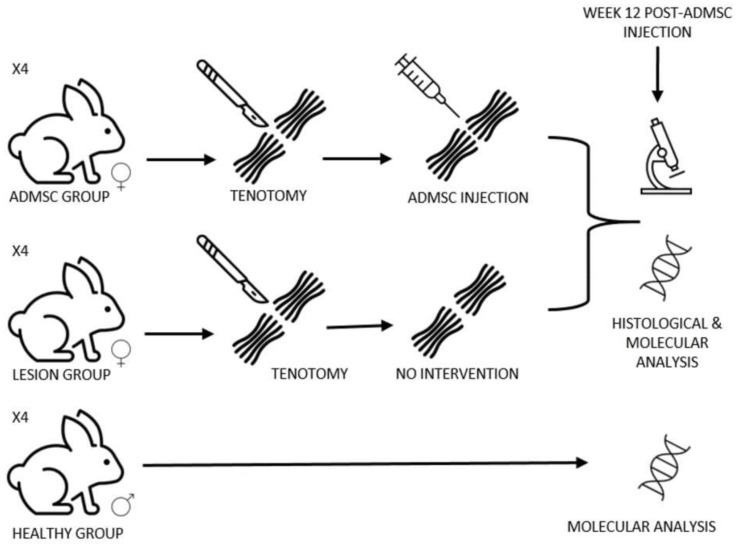
Workflow scheme. Eight female New Zealand white rabbits in which tenotomy was performed, were divided into two groups (four animals per group). The ADMSC group received male ADMSCs after tenotomy, whereas the lesion group remained untreated (no intervention). At Week 12, tissue biopsies of both groups were taken in order to perform histological and molecular analysis. An additional healthy group of four male rabbits was simultaneously analyzed as a control.

In order to evaluate the efficacy of ADMSCs to accelerate tendon tissue repair, 1 × 10^6^ male flow-cytometry-characterized ADMSCs obtained from the liver falciform ligament were injected into female rabbits directly in the lesion (ADMSC group) and compared to the untreated group (lesion group) 12 weeks later via molecular and histological assays. A basic scheme of the experiments is depicted in Figure 1.

### 3.1. Flow Cytometry Analysis

**Figure 2:**
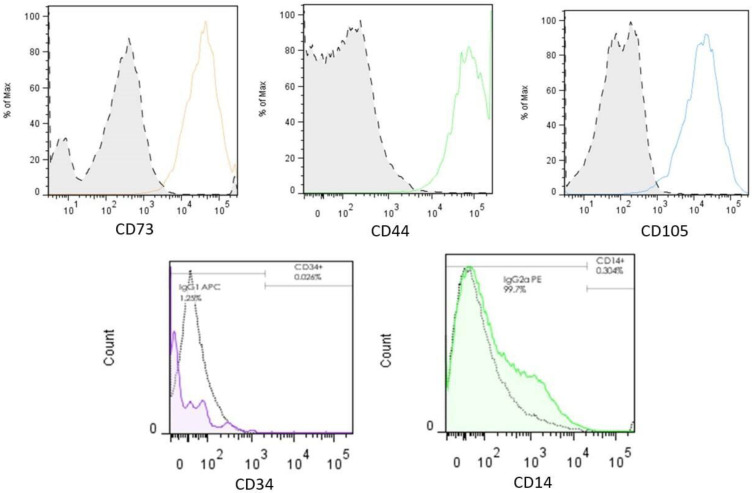
Flow cytometry analysis. Adherent cells that showed MSC morphology were characterized using specific markers for stem cells according to the International Society for Cellular Therapy (ISCT). Negative controls of each antibody’s own isotype were included to rule out nonspecific signals and unlabeled cells (dotted line curves). CD73, CD44, and CD105 stained datasets were found positive after overlay, whereas CD34 and CD14 stained datasets were negative, thus discarding a hematopoietic origin of the sampled cells and suggesting an MSC phenotype.

Prior to administration into the ADMSC group, characterization of the falciform ligament-obtained cells was performed with flow cytometry. Immunophenotyping of the ADMSC group revealed no surface markers associated with hematopoietic cells (CD34 and CD14), making possible the distinction of cells with characteristics of a mesenchymal origin, as revealed by the CD73+, CD44+, and CD105+ markers previously accepted by the International Society for Cellular Therapy (ISCT) [25] and found in the cells subjected to study (Figure 2).

### 3.2. Molecular Analysis

**Figure 3:**
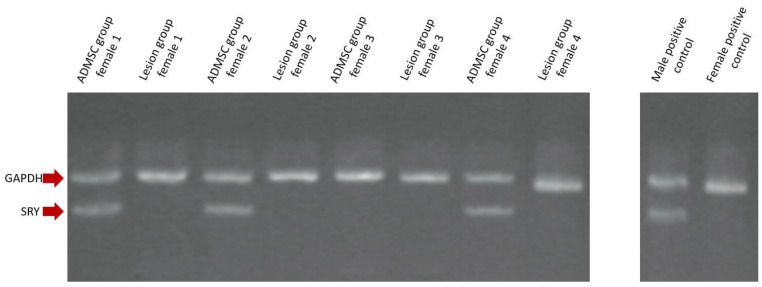
Identification of SRY. The sex-determining region Y (SRY) gene, which has an amplified product of 241 bp was identified in 3 out of 4 female rabbits of the ADMSC group and absent in all the members of the lesion group. GAPDH (287 bp) was used as an internal control and was identified in all members as expected. Male and female controls are also shown.

To better differentiate between self or implanted ADMSCs, male ADMSCs were detected among host female cells via the SRY gene detection, located in the Y chromosome. PCR was performed in both ADMSC and lesion groups with the aim of detecting the aforementioned SRY gene. The SRY gene was identified in the tissue obtained from three out of four sacrificed rabbits in the ADMSC group but was undetected in any of the members of the lesion group, indicating that the ADMSCs migrated and remain viable at the site of injury at Week 12 post-cell administration (Figure 3).

**Figure 4:**
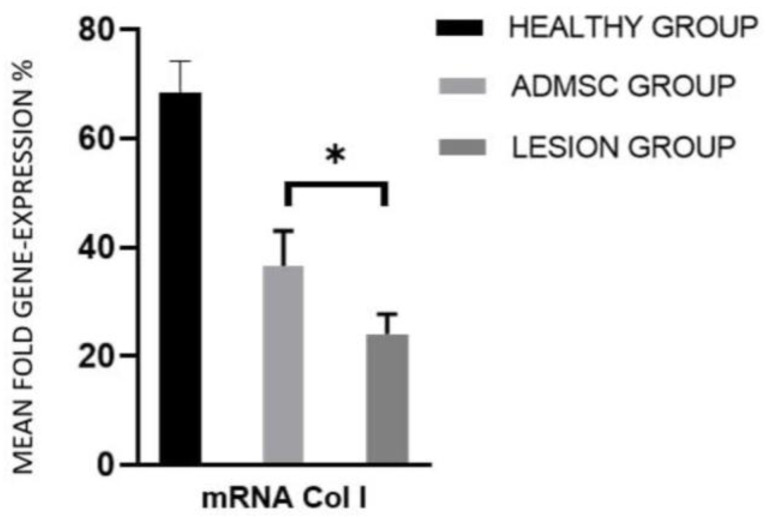
Mean fold gene expression % for collagen I relative to GAPDH in healthy, ADMSC, and lesion groups determined by quantitative RT-PCR. Standard error of the mean (SEM) is also depicted. * *p* < 0.05.

Quantitative RT-PCR was performed in all the animal models tested for SRY gene presence in order to evaluate the expression of collagens, which are reliable markers of repair activity. The expression levels were normalized to GAPDH expression and reported as mean fold gene expression percentages relative to this housekeeping gene, called mean expression levels hereinafter in the text (Table 2 and Table 3). The mean expression levels for collagen I in the ADMSC and lesion groups were 36.6% and 24.1%, respectively. This indicates that the cells derived from adipose tissue were able to stimulate a greater expression of type I collagen, which is critical in the structure of the Achilles tendon (Figure 4).

**Figure 5:**
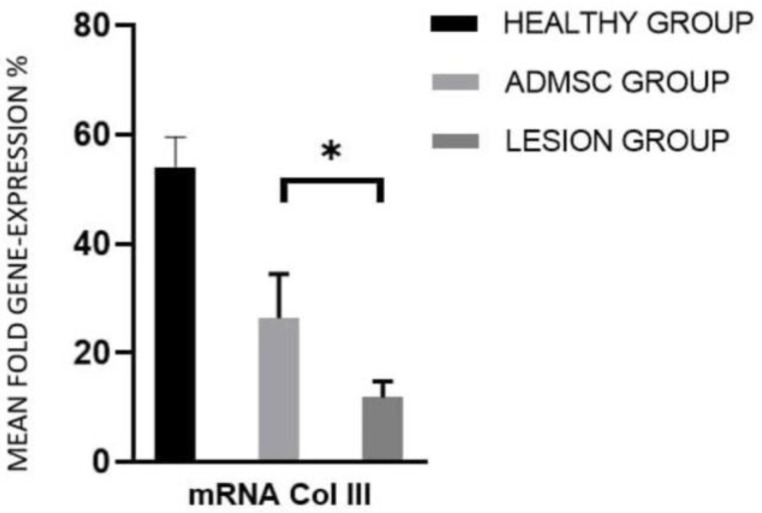
Mean fold gene expression % for collagen III relative to GAPDH in healthy, ADMSC, and lesion groups determined by quantitative RT-PCR. Standard error of the mean (SEM) is also depicted. * *p* < 0.05.

On the other hand, the mean expression levels for collagen III in the ADMSC and lesion groups were 26.3% and 11.9%, respectively. This indicates that the applied cells were able to stimulate a higher formation of type III collagen, which directly influences the arrangement of type I collagen fibers, thus not correlating with the histopathological observations (Figure 5). The expression ratio between the ADMSC group and the lesion group was 1.5 and 2.2 for collagen I and collagen III, respectively (Table 4). Ratios of collagen III/collagen I mean expression reported as a parameter in some studies [26] were 0.7 for the ADMSC group and 0.5 for the lesion group.

### 3.3. Histological Analysis

Once we observed differences in collagen expression between the ADMSC and lesion groups, we proceeded to identify any differences at the tissue level histologically.

**Figure 6:**
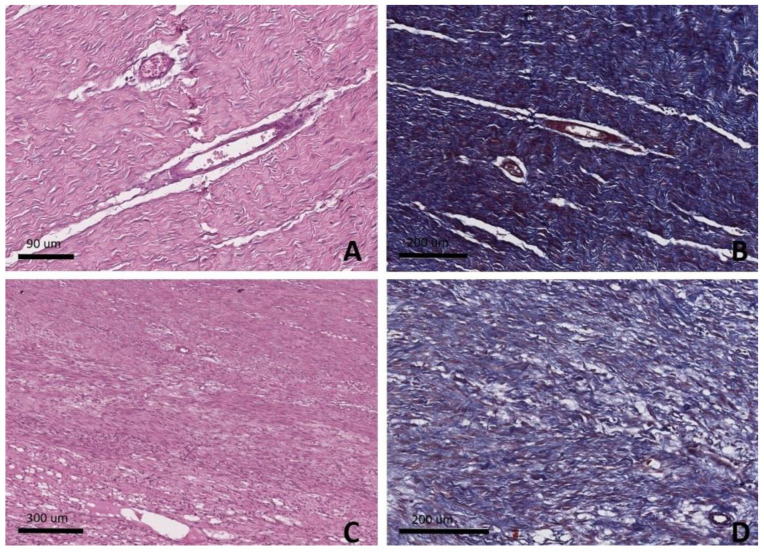
Histopathology. (**A**) H&E staining of ADMSC group. Vascular neoformation is observed along with moderately disorganized fibers and formation of blood vessels with cellular aggregates. (**B**) Masson’s trichrome of ADMSC group. Vascular neoformation is observed along with moderately disorganized fibers. (**C**) H&E staining of lesion group. Areas of inflammatory infiltrate are observed along with abundant areas of adipose infiltration between the collagen fibers, few blood vessels, and necrotic foci. (**D**) Masson’s trichrome of lesion group. Areas of inflammatory infiltrate are observed along with abundant areas of adipose infiltration between the collagen fibers, few blood vessels, and necrotic foci.

After Masson’s trichrome and H&E staining, the results of the histopathological analysis graded by the GHBCS [18] indicated no statistically significant changes in collagen fiber orientation or angiogenesis in the ADMSC group compared to the lesion group. No cartilage formation was detected in any of the studied groups (Figure 6) (Table 5).

## 4. Discussion

Acute Achilles-tendon injuries are relatively common and present a slow, complex, and inefficient natural repair process due to their intrinsic low cellularity and low vascularity. This represents a clinical challenge for orthopedists looking for the best treatment option. The most frequent causes of tendon injuries are mainly associated with the practice of sports, aging, tendinopathies, hypothyroidism, hypertension, diabetes mellitus, arthropathies, corticosteroids, and vitamin C deficiency, among other causes [19]. Biological approaches using mesenchymal stem cells, such as those derived from adipose tissue, pose a potential treatment option. Although there is currently little evidence for their clinical therapeutic use, it is thought that the efficacy of ADMSCs in regenerative medicine may be due to the release of growth factors and cytokines, as well as their immunomodulatory effects via extracellular vesicles (EVs) to promote certain cellular events associated with tissue repair [20,21,22].

Moreover, there are only a few studies using animal models to determine whether ADMSCs injected in situ are capable of improving histological signs of tendon healing.

In our study, using an animal model of a larger evolutionary scale, saline solution as a means of cell transport, and the SRY gene to track male rabbit ADMSCs directly administered in the lesion of female rabbits, we observed the presence of the stem cells at Week 12 post-treatment in three out of four treated rabbits in the ADMSC group, both with no presence in the lesion group. The reason for the absence of ADMSCs at Week 12 in a unique animal model in the ADMSC group is unknown; however, it could be speculated that a low level of cell homing or permanence could cause this result. Interestingly, this apparent SRY-negative animal model was positive for an increment in both collagen I and collagen III, similar to the rest of the animal models in the same group. This particular observation could suggest a future experiment to interrogate the possibility of tissue healing or at least augmented collagen expression in late stages of repair in the absence of ADMSCs via prolonged induction or host stem cells recruiting without the permanence of the originally implanted ADMSCs. In addition, no significant histopathological changes in collagen formation were observed in the group that received ADMSCs compared to the untreated group at Week 12 post-administration. Indeed, the absence of significant histological changes, including increases in collagen fibers, has previously been suggested despite mechanical improvement of Achilles-tendon injuries 3 weeks after administration of hydrogel-containing ADMSCs in rats [23]. These findings, when contrasting with ours, could indicate that the histological findings are present after 12 weeks post-treatment or that the histological analysis is not a useful parameter to measure the functional improvement of the tissue in this particular case. Another study reported an improvement in the organization of collagen fibers and morphometric nuclear parameters at Week 3 post-treatment, but did not find statistically significant differences with the control at Weeks 6 and 12 in the same model used in the present study, which seems to consolidate our results at Week 12 while contrasting with the research that establishes an improvement at Week 3 as previously mentioned [24]. The discrepancies reported here appear to be largely due to differences in the vehicle used, the technique by which the tendon was damaged, the number of cells administered, and the animal model used.

The mean expression level for collagen I in the group of animals that received treatment with ADMSCs was 36.6%, in contrast with the 24.1% of the lesion group. Moreover, the expression ratio between these groups was 1.5 for collagen I. This indicates that the cells derived from adipose tissue were able to stimulate a greater expression of type I collagen, which is an important component of the Achilles-tendon structure. Mean expression level for collagen III in the group of animals that were treated with ADMSCs was 26.3%. On the other hand, the lesion group achieved 11.9% of mean collagen III expression level. The expression ratio between these groups was 2.2 for collagen III. This indicates that the applied cells were able to stimulate a higher formation of type III collagen, which directly influences the arrangement of type I collagen fibers.

Several histologic phases of tendon repair have been previously suggested [27]. These findings establish that during Weeks 12 to 14, the repair continues with a return to the normal concentration and proportion of collagens, with predominance mainly of type I collagen. This observation could indicate that a greater expression of collagen I and III could be expected in times shorter than 12 weeks, as observed in the present study.

Likewise, in the present work, using the SRY gene as a method of identification of ADMSCs, we were able to determine the presence of the cells applied in the area of the lesion at Week 12, which is consistent with previous studies that show the presence of the cells in that temporality by microscopy, which complements our findings [24].

Moreover, several groups also evaluate the effects of ADMSCs with collagens expression ratios. In a recent study, ADMSCs were assayed in a collagenase-induced rat model of tendinopathy. In this study, using quantitative RT-PCR to measure the ratio of expression of collagens in injured tendons, the ratio of type III collagen to type I collagen (collagen III/collagen I) was found to be significantly lower in the ADMSC group than in the lesion group at Week 12. Furthermore, this proportion decreased over time in the treated group with ADMSCs, while it increased over time in the lesion group [26]. This result contrasts with ours in which the inverse was observed. It could be possible that the evaluation of this ratio is not a useful parameter or simply does not correlate with the tissue repair status. Instead, according to what was seen in our work, we suggest that the augment of both types of collagens represents a better parameter of tendon healing; however, this suggestion remains to be confirmed. Moreover, as previously mentioned, most of the discrepancies could be at least partially due to the different animal models employed in different studies. Nevertheless, since we employed an animal model, which is of a higher evolutionary scale than rats and with a greater demand in the biomechanics of its Achilles tendon that could better resemble human tendinous tissue, we believe that our results represent a valuable observation to stem cell therapy applied to human patients. However, limitations in the current study such as small sample size and absence of analyses of other important markers such as scleraxis and tenomodulin can be covered in the future to support our findings.

## 5. Conclusions

The findings of the present study suggest that the application of ADMSCs in Achilles-tendon injuries may favor structural repair by augmenting the expression of collagen I and III genes even though structural changes were not observed as in previous work of several research groups. It requires longer observation times to determine the completeness or extension of tissue repair, as well as studies that analyze the stress modulus in order to estimate if the pathological findings are consistent with resistance to biomechanics in the animal model employed.

## 6. Patents

A patent was not generated from this research work.

## Figures and Tables

**Table 1:** Flow cytometry targets and antibodies.

Target	Antibody	Fluorophore	Brand
	Mesenchymal		
CD105	Anti-CD105 IgG1, κ	FITC	Serotec
CD73	Anti-CD73 IgG1, κ	PE	Invitrogen
CD44	Anti-CD44 IgG2a, κ	PE	Chemicon
	Hematopoietic		
CD14	Anti-CD14 IgG2a, k	PE	Abd Serotec
CD34	Anti-CD34 IgG1, κ	PE	BD Biosciences
Control	IgG2a, κ Isotype	PE	BioLegend
Control	IgG1, κ Isotype	PE	BioLegend

**Table 2:** Collagen I gene expression.

	Collagen I		
	Healthy Group	ADMSC Group	Lesion Group
Mean Fold Gene Expression	0.6	0.3	0.2
Mean Fold Gene Expression %	68.5%	36.6%	24.1%

**Table 3:** Collagen III gene expression.

	Collagen III		
	Healthy Group	ADMSC Group	Lesion Group
Mean Fold Gene Expression	0.5	0.2	0.1
Mean Fold Gene Expression %	54%	26.3%	11.9%

**Table 4:** Gene expression ratios.

	Collagen I	Collagen III
ADMSC Group/Lesion Group Expression Ratio	1.5	2.2

**Table 5:** Histopathology results with GHBCS.

	ADMSC Group (Mean with IQR) (*n* = 4)	Lesion Group (Mean with IQR) (*n* = 4)	*p*
Collagen	1.5 (1–2)	2 (2–3)	0.13
Angiogenesis	1 (0–1)	0 (0–0.75)	0.23
Cartilage	0 (0–0)	0 (0–0)	0.99

## Data Availability

Not applicable.
